# Extensor tendon rupture and preoperative mri confirmations of suture anchor prolapse: a case report and literature review

**DOI:** 10.1186/s12891-024-07476-0

**Published:** 2024-05-04

**Authors:** Ahmad Alhaskawi, Haiying Zhou, Yanzhao Dong, Sohaib Hasan Abdullah Ezzi, Xiaodi Zou, Zhou Weijie, Fangyu Yi, Sahar Ahmed Abdalbary, Hui Lu

**Affiliations:** 1 Department of Orthopedics, The First Affiliated Hospital, Zhejiang University, #79 Qingchun Road, Hangzhou, Zhejiang Province 310003 P. R. China; 2 Department of Orthopedics of the Third Xiangya Hospital, Central South University, Tongzipo Rd, Changsha, Hunan 410083 China; 3 The Second Affiliated Hospital of Zhejiang Chinese Medical University, Zhejiang Province, 310003 Hangzhou, P. R. China; 4 Department of Orthopaedics, Joint Service Assurance Force 903 Hospital, Airport Road, Shangcheng District, Hangzhou City, Zhejiang Province 310053 P.R. China; 5 The First School of Clinical Medicine, Zhejiang Chinese Medical University, #548 Binwen Road, Hangzhou, Zhejiang Province 310053 P.R. China; 6 Department of Orthopedic Physical Therapy, Faculty of Physical Therapy, Nahda University in Beni Suef, Beni Suef, Egypt

**Keywords:** Suture anchor prolapse, Distal phalanx fracture, Tendon adhesion, Bone anchor, Mallet finger, Hand

## Abstract

**Background:**

While suture anchors are widely used in medical procedures for their advantages, they can sometimes lead to complications, including anchor prolapse. This article presents a unique case of suture anchor prolapse at the base of the distal phalanx of the little finger after extensor tendon rupture reconstruction surgery.

**Case presentation:**

A 35-year-old male, underwent extensor tendon rupture reconstruction using a non-absorbable suture anchor. After seven years the patient visited our outpatients complaining of stiffness, pain, and protrusion at the surgical site. Initial X-ray imaging suggested suggesting either a fracture of the distal phalanx or tendon adhesion but lacked a definitive diagnosis. Subsequent magnetic resonance imaging (MRI) revealed bone connectivity between the middle and distal phalanges with irregular signal shadow and unclear boundaries while maintaining a regular finger shape. MRI proved superior in diagnosing prolapsed suture anchors, marking the first reported case of its kind. Surgical intervention confirmed MRI findings.

**Conclusions:**

Suture anchor complications, such as prolapse, are a concern in medical practice. This case underscores the significance of MRI for accurate diagnosis and the importance of tailored surgical management in addressing this uncommon complication.

## Introduction

Mallet finger, a condition resulting from trauma to the fingertip, presents as a drooping distal joint due to damage to the extensor tendon. Non-surgical options, such as splinting to maintain joint position, physical therapy for rehabilitation, and the RICE protocol for symptom management are often effective in mild cases. For severe tendon damage or fractures, surgical interventions like Open Reduction and Internal Fixation (ORIF), tendon repair, or joint fusion may be necessary [1]. Advanced techniques, including extensor tendon suture anchor fixation to the base of the distal phalanx, are used in severe cases. This procedure achieves precision by securing the damaged extensor tendon to the bone with suture anchors, promoting optimal healing, and restoring the proper alignment of the distal joint [2, 3]. However, complications may arise in cases of unsuccessful treatment or delayed intervention. Persistent extensor tendon insufficiency can lead to conditions such as DIP (distal interphalangeal) joint flexion contracture, characterized by an inability to fully extend the affected joint. Additionally, swan-neck deformity, marked by hyperextension of the proximal interphalangeal (PIP) joint and flexion of the DIP joint, can occur [4–6]. Postoperative care involves immobilization followed by a tailored rehabilitation program to restore functionality. Early diagnosis and an individualized approach to treatment are essential for optimal recovery in mallet finger cases [1, 7, 8]. The suture anchor is a specialized medical implant used in orthopedic surgeries to attach soft tissues, such as tendons and ligaments, to bone [9]. However, the use of suture anchors may result in several potential complications. For instance, an inflammatory response may occur, leading to osteolysis after surgery. Studies have documented significant bony defects in the distal phalanx at the suture anchor insertion site, as seen on X-rays in some cases. Additionally, this inflammatory reaction can cause adhesion of the flexor digitorum profundus (FDP) tendon to the distal phalanx. It is crucial to be aware of these possible complications when considering the use of suture anchors and to carefully monitor patients postoperatively for any signs of adverse reactions [10–14].

This article reports a case where an MRI eventually detected suture anchor prolapse. Despite conducting a physical examination and obtaining X-ray images, the initial assessment was misleading and suggested either a mallet finger or tendon adhesion.

## Case presentation

**Fig. 1:**
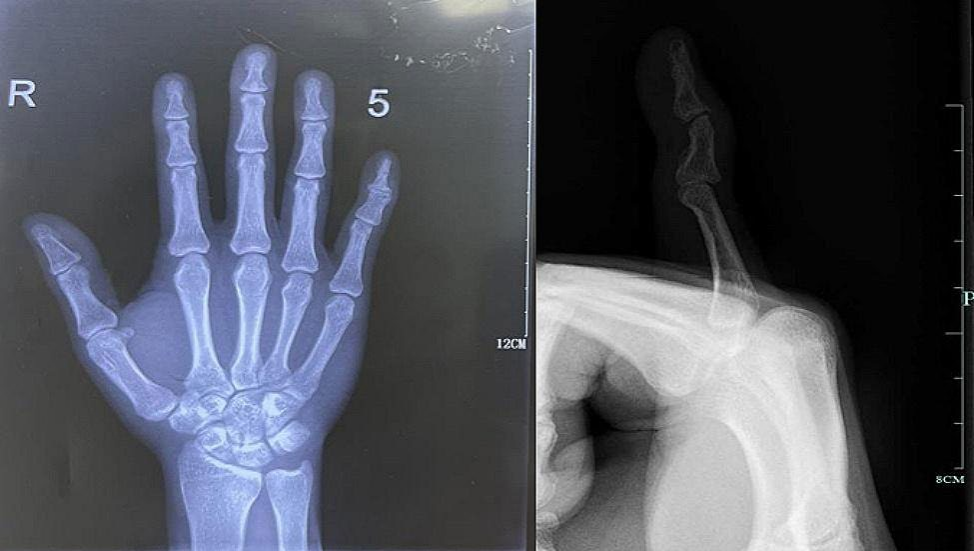
X-ray of the right hand presenting a malunion fracture block at the middle and distal phalanges of the right little finger

A 35-year-old male patient previously undergone extensor tendon rupture reconstruction surgery on his right little finger at another hospital. During the procedure, a suture anchor was placed at the base of the distal phalanx. Seven years post-surgery, the patient visited our department, complaining of pain, and stiffness in his finger. Notably, he reported no post-surgical trauma. Physical examination showed an inability to achieve full dorsal extension, but there was no disturbance of circulation or swelling. Mild redness and signs of infection were observed, along with a noticeable protrusion at the surgical site. An X-ray was performed, revealing an anomaly at the base of the distal phalanx of the little finger, raising suspicions of either a distal phalanx fracture or tendon adhesion (Fig. 1).

**Fig. 2:**
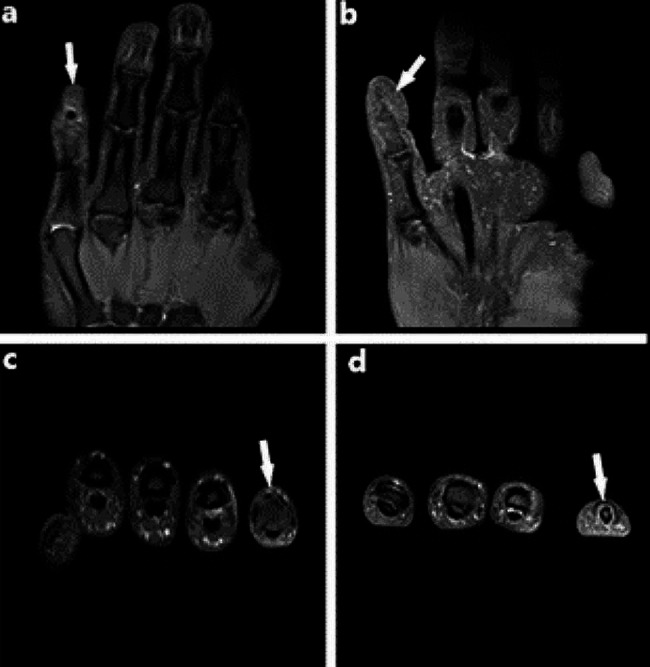
Comparing between MRI before and after the surgery. **(a, c)** preoperative images, show a foreign body, which did not rupture the extensor tendon, and a poor bone connectivity of the middle and distal phalanges, with patchy (lipid suppression) high signal shadow and unclear boundary at distal phalanx of the little finger(narrows). **(b, d)** postoperative images, present no foreign body shadow

**Fig. 3:**
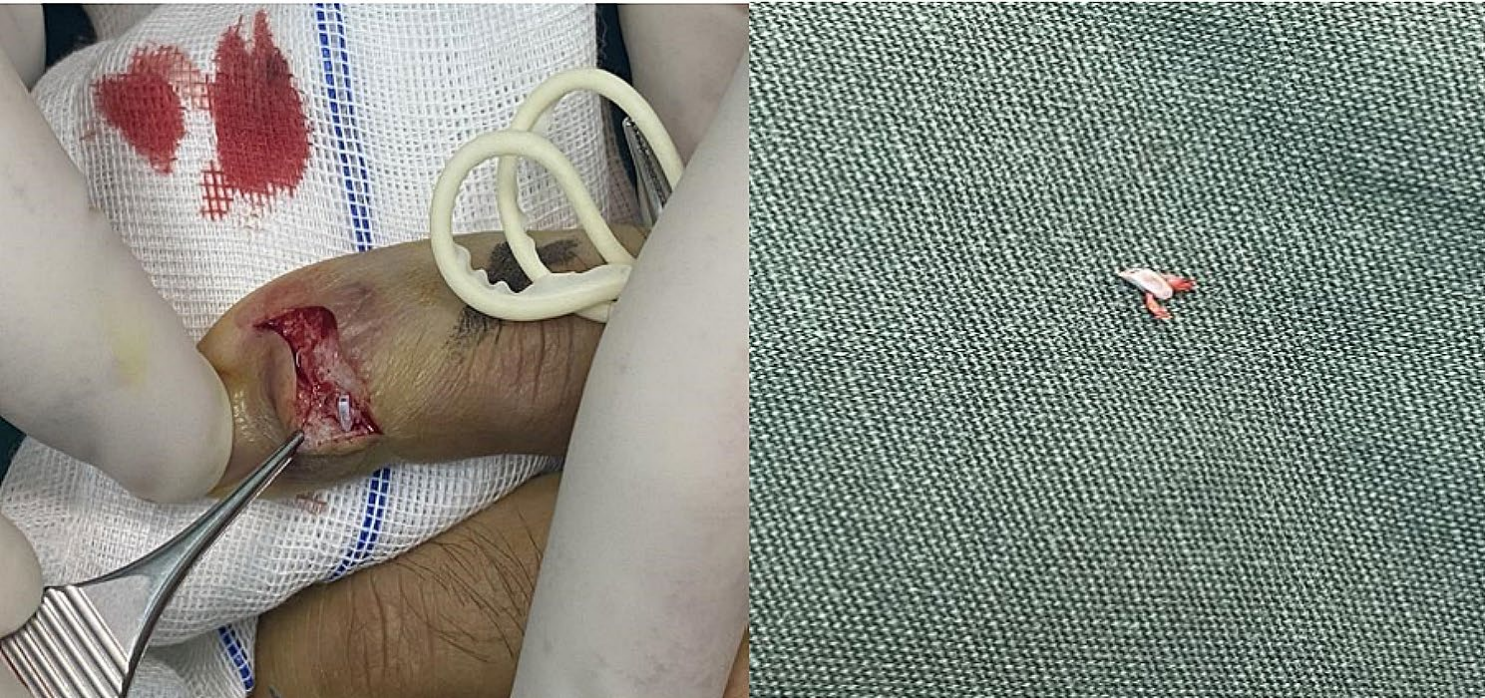
Suture anchor prolapse at the base of distal phalanges of the right little finger, which could be misdiagnosed as an osteophyte or tendon adhesion

Following further investigation, an MRI was performed, revealing abnormal alterations in both the middle and distal phalanges of the right little finger. These changes were characterized by compromised bone connectivity, irregular areas of patchy high signal shadows (notably with lipid suppression), and indistinct boundaries. Notably, the bone morphology of the other right fingers appeared normal, displaying no discernible abnormal signals. Additionally, the joint surfaces were smooth, with no abnormalities detected in the surrounding soft tissues (Fig. 2a, c). Therefore, the patient underwent surgery, where a significant amount of scar tissue around the inserted suture anchor. Following the removal of the scar tissue, it was discovered that the tail of the anchor was located in the center of the extensor tendon, close to the subcutaneous area, and protruding. Attempts to clamp the anchor directly through the tail were unsuccessful. After the base is enlarged with an electric drill, the anchor was completely removed. The bone defect was reinforced by artificial bone grafting and re-repair of the extensor tendon was performed (Fig. 3). Our patient was diagnosed with anchor prolapse, which was clearly visible on magnetic resonance MRI.

**Fig. 4:**
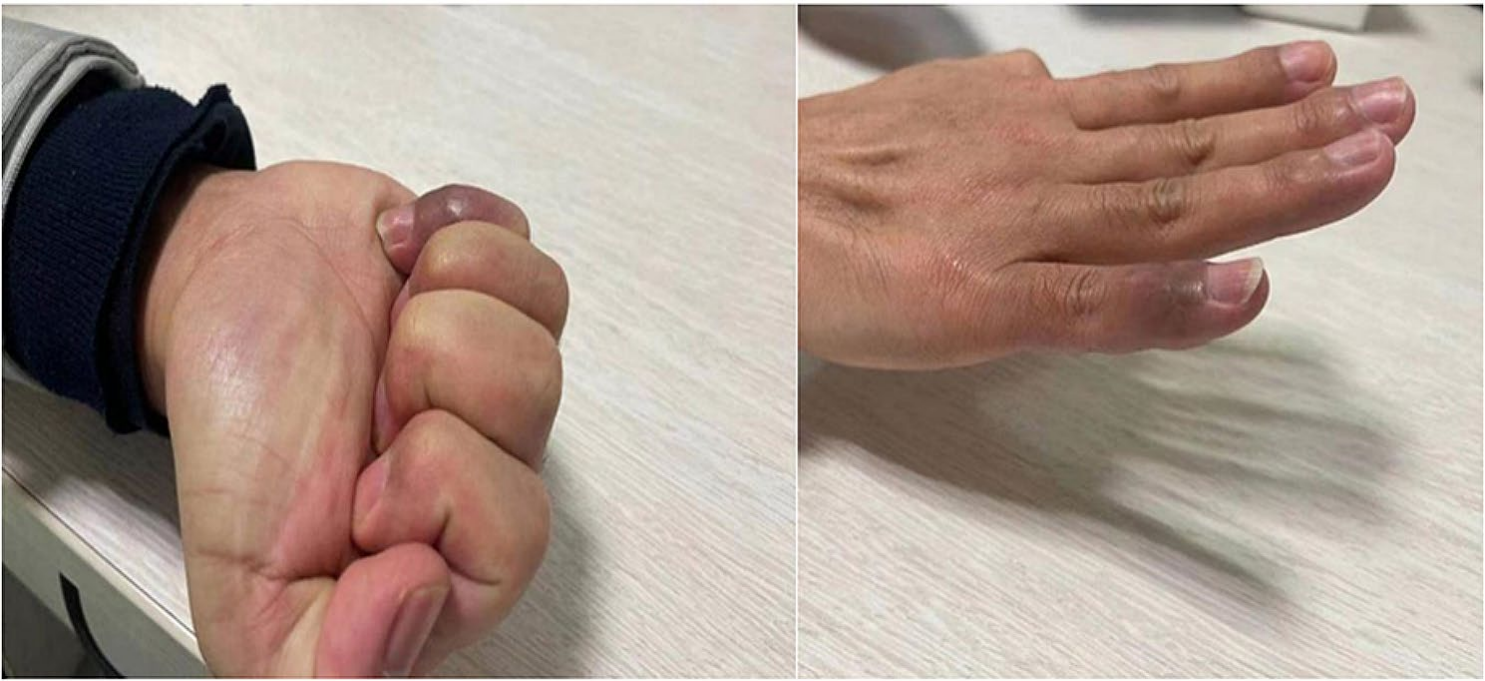
Second month after removing the prolapsed suture anchor, noticeable improvement of the movement of the right little finger

A finger-cap fixation was applied for one month, accompanied by the administration of oral antibiotics. The stitches were subsequently removed two weeks post-surgery. Functional exercises began after two weeks. At one month follow-up, the flexion and extension function of the distal segment showed improvement compared to its pre-operative state, and the nearly full range of motion was observed at the second-month follow-up (Fig. 2b, d, and Fig. 4).

## Discussion

Suture anchors represent a pivotal advancement in modern surgical techniques, particularly in the field of orthopedic and sports medicine. Their primary function is to enable the secure attachment of soft tissues, such as ligaments and tendons, to bone, a task that traditional suturing methods may not adequately accomplish. These anchors, typically composed of metal or biocompatible polymers, are meticulously designed for insertion into the bone, providing a robust and reliable anchorage point for sutures. This technology is especially beneficial in areas subjected to high stress and movement, such as shoulder, hand, and knee joints, where it ensures a stable and enduring tissue-to-bone healing process. The utilization of suture anchors has been instrumental in reducing recovery time, minimizing postoperative complications, and enhancing the overall success rates of orthopedic surgeries. Their versatility and effectiveness in various surgical contexts underscore their significance in contemporary medical practice [15, 16].

A key factor in the successful use of suture anchors is the selection of the appropriate anchor. This choice involves considering the size and material of the anchor, which may vary from metallic to bioabsorbable, depending on the patient’s condition and the specific surgical requirements. Moreover, the insertion technique is paramount [17]. The insertion process involves loading the suture into the anchor implant, placing it into a pre-drilled bone hole, and then applying tension by pulling on the free suture ends. This is followed by securing the suture end to suture cleats for stabilization, as described by Kevi Es Neison and Joodan Ei Foof [18]. According to Johnstone and Karuppiah [19], a proper technique involves using a guide wire or pilot hole for precise placement and ensuring that the anchor is inserted at an optimal angle to maximize pull-out strength. Additionally, it is vital to avoid overtightening the anchor, as this can lead to bone damage or anchor loosening [19]. To facilitate easier insertion of suture anchors, surgeons may employ various techniques. Proper drilling, using the correct drill bit size and depth, is crucial. Depth gauges can aid in achieving the correct depth and prevent drilling too deep or shallow. Enhanced visualization techniques, such as arthroscopic or imaging methods, are also recommended for precise placement, especially in less accessible surgical areas. Additionally, the use of ergonomic instruments designed for anchor insertion can improve the surgeon’s control and accuracy during the procedure [17, 20, 21]. However, the use of suture anchors is not without its disadvantages. The risk of prolapse or migration of the anchor remains a concern, particularly if the placement technique is not precise. There is also a potential risk of infection, a common issue with any surgical implant. Cost is another factor, as suture anchors tend to be more expensive than traditional suturing methods. Lastly, the effectiveness of suture anchors relies heavily on the surgeon’s expertise and experience, indicating a significant learning curve for optimal use [22, 23].

Suture anchor prolapse, where the anchor dislodges or moves from its initially intended position, can occur for several reasons, and understanding these factors is crucial for preventing such complications. One of the primary reasons for suture anchor prolapse is poor bone quality. In patients with osteoporosis or other conditions that weaken the bone, the anchor may not secure properly, leading to a higher risk of prolapse. This issue is particularly significant in elderly patients or those suffering from diseases that affect bone density. Additionally, the use of an anchor that is too small or not suitable for the specific bone density can also lead to inadequate fixation strength, increasing the likelihood of prolapse. It’s essential to select an anchor size and type that is compatible with the patient’s bone quality and the specific requirements of the surgical procedure [24, 25]. In addition, incorrect placement of the suture anchor is another significant factor contributing to prolapse. If the anchor is not placed at the correct depth or angle, it may not hold securely in the bone. Placement that is too superficial or in an area of the bone with less density can compromise the anchor’s stability. Furthermore, overloading the anchor by applying excessive tension to the suture or using it in a high-stress area without adequate support can lead to the failure of the anchor. Surgeons must ensure that the anchor is placed correctly and that the suture is tensioned appropriately to prevent such issues [22, 24]. Suboptimal surgical techniques can also lead to anchor prolapse. Inadequate preparation of the anchor site, such as not pre-drilling a hole to the appropriate size or not cleaning the hole of debris before anchor insertion, can affect the anchor’s grip in the bone. Precision in surgical technique and thorough site preparation are therefore essential [26, 27]. Furthermore, Patient factors play a significant role as well. Activities that place excessive stress on the area soon after surgery or non-compliance with postoperative restrictions can contribute to anchor prolapse. Additionally, conditions that affect healing, such as diabetes or smoking, may also increase the risk [22, 28, 29]. Suture anchor prolapse can often result in a range of symptoms, such as pain, stiffness, and mass projection at the prolapsed site, which can be similar to other conditions such as distal phalanx fracture and tendon adhesion. X-ray imaging of anchor prolapse typically reveals an avulsion fragment at the insertion site of the common extensor tendon on the distal phalanx at the distal interphalangeal joint, which may resemble a mallet finger type of distal phalanx fracture or tendon adhesion [16]. Distal phalanx fractures are frequently encountered in both emergency departments and outpatient clinics. Mallet finger deformities typically result from an avulsion injury to the terminal tendon of the distal phalanx, which leads to the detachment of a bone fragment from the insertion of the terminal tendon [30]. X-ray imaging typically reveals an avulsion fragment at the base of the common extensor tendon, indicating a mallet finger injury. Notably, a high proportion of mallet finger injuries present as isolated tendon injuries without associated avulsion fractures [31]. In addition, tendon adhesion, characterized by the adhesion of tendons to surrounding tissues and the loss of range of movement, can be diagnosed at the distal phalanx of the little finger by X-ray imaging and during the physical examination [32–35]. Tendon adhesion is a reported complication in up to 20% of patients with tendon injuries [32–35].

Therefore, our case presented the benefit of MRI in diagnosing and locating the suture anchor prolapse at the distal phalanx of the little finger after, which had been misdiagnosed by X-ray imaging. The MRI scan revealed unusual changes in both the middle and distal phalanges of the right little finger. These changes were characterized by compromised bone connectivity, irregular areas of patchy high signal shadows (notably with lipid suppression), and indistinct boundaries, conclusively confirmed the presence of suture anchor prolapse. The MRI finding was confirmed through surgical intervention. In contrast, conditions such as mallet finger and tendon adhesion are typically diagnosed through clinical examination, ultrasound, and X-ray imaging.

## Conclusion

This article presents a case in which the eventual identification of suture anchor prolapse was made through MRI, revealing a discrepancy from the initial assessment. Despite a thorough physical examination and the acquisition of X-ray images, the initial evaluation was misleading, pointing towards potential diagnoses such as a mallet finger or tendon adhesion. An inaccurate diagnosis may result in delayed or inappropriate treatment, underscoring the importance of careful consideration of radiological findings. Therefore, MRI has proven to be an invaluable diagnostic tool for detecting prolapsed suture anchors.

## Data Availability

No datasets were generated or analysed during the current study.

## Abbreviations

MRI: Magnetic resonance imaging
ORIF: Open Reduction and Internal Fixation
DIP: (distal interphalangeal)
PIP: Proximal interphalangeal joint
FDP: Flexor digitorum profundus
